# Supplementary material for: An open-source framework for large-scale, flexible evaluation of biomedical text mining systems
Source: J Biomed Discov Collab. 2008 Jan 29;3:1. doi: 10.1186/1747-5333-3-1 (PMC2276192; doi:10.1186/1747-5333-3-1)
Supplement: Additional file 1 — Evaluation platform source code. The additional file contains the software evaluation framework discussed in this paper. The most current release can be downloaded from SourceForge.net [1]. See the accompanying README file for instructions on installation and use. Also included in the distribution is a collection of UIMA wrappers for some commonly used BioNLP tools and annotated corpora. [file 1747-5333-3-1-S1.zip › bionlp_uima_repository-1.0.1/docs/api/src-html/edu/uchsc/ccp/util/nlp/mention/comparison/IdenticalSlotMentionComparator.html]

```
001    package edu.uchsc.ccp.util.nlp.mention.comparison;
002    
003    import edu.uchsc.ccp.util.nlp.annotation.comparison.SpanComparator;
004    import edu.uchsc.ccp.util.nlp.mention.Mention;
005    import edu.uchsc.ccp.util.nlp.mention.SlotMention;
006    
007    public class IdenticalSlotMentionComparator extends IdenticalMentionComparator implements SlotMentionComparator {
008    
009        /**
010         * Compare two mentions. They must be SlotMentions or else an UnsupportedOperationException is thrown. In order to be identical (and return 0),
011         * they must have the name mention name, and the identical slot values
012         */
013        @Override
014        public int compare(Mention mention1, Mention mention2, SpanComparator spanComparator, int maximumComparisonDepth, int depth) {
015            if ((mention1 instanceof SlotMention) & (mention2 instanceof SlotMention)) {
016                return compare((SlotMention) mention1, (SlotMention) mention2, spanComparator, maximumComparisonDepth, depth);
017            } else {
018                throw new ClassCastException("Cannot support comparison between a SlotMention and a non-SlotMention");
019            }
020        }
021    
022        /**
023         * Compare two SlotMentions. In order to be identical (and return 0), they must have the same mention name, and the identical slot values
024         * 
025         * @param slotMention1
026         * @param slotMention2
027         * @param maximumComparisonDepth
028         * @param depth
029         * @param spanComparator
030         * @param compareLinkedAnnotationSpans
031         * @return
032         */
033        public int compare(SlotMention slotMention1, SlotMention slotMention2, SpanComparator spanComparator, int maximumComparisonDepth,
034                int depth) {
035            if (slotMention1.getSlotValues().size() == slotMention2.getSlotValues().size()) {
036                /* quick check to make sure each slot mention has the same number of slot values */
037                if (hasEquivalentMentionNames(slotMention1, slotMention2)) {
038                    for (Object slotValue : slotMention1.getSlotValues()) {
039                        boolean objectHasMatch = false;
040                        for (Object svToCompare : slotMention2.getSlotValues()) {
041                            if (slotValue.equals(svToCompare)) {
042                                objectHasMatch = true;
043                            }
044                        }
045                        if (!objectHasMatch) {
046                            /* if they do not match, then simply return the comparison between their single line representation strings */
047                            return slotMention1.getSingleLineRepresentation().compareTo(slotMention2.getSingleLineRepresentation())
048                                    * MULTIPLIER;
049                        }
050                    }
051                    /* if we get to this point, then all slot values have a match, so return 0 */
052                    return 0;
053                } else {
054                    /* the sm's have different mention names */
055                    return slotMention1.getSingleLineRepresentation().compareTo(slotMention2.getSingleLineRepresentation()) * MULTIPLIER;
056                }
057            } else {
058                /* the sm's have unequal number of slot values */
059                return slotMention1.getSingleLineRepresentation().compareTo(slotMention2.getSingleLineRepresentation()) * MULTIPLIER;
060            }
061    
062        }
063    
064    }
```
